# Pneumococcal serotype distribution and diagnostic yield of serotype-specific urinary antigen detection in adults with CAP in Switzerland, 2016–2021: a prospective cohort study

**DOI:** 10.1186/s12879-026-14493-y

**Published:** 2026-09-21

**Authors:** Samuel Etienne, Werner C. Albrich, Mathias W. Pletz, Marcus Panning, Vivian Suarez Domenech, Frank Eberhardt, Grit Barten-Neiner, Daiana Stolz

**Affiliations:** 1 Clinic of Respiratory Medicine and Pulmonary Cell Research, University Hospital of Basel, Basel, Switzerland; 2 Division for Lung & Airway Research, Institute of Environmental Medicine, Karolinska Institutet, Stockholm, Sweden; 3 Division of Infectious Diseases, Infection Prevention and Travel Medicine, HOCH Health Ostschweiz, Cantonal Hospital St. Gallen, St. Gallen, Switzerland; 4 Institute for Infectious Diseases and Infection Control and Center for Sepsis Care and Control, Jena University Hospital, Jena, Germany; 5 German Centre for Lung Research, DZL, Biomedical Research in Endstage and Obstructive Lung Disease Hannover (BREATH), Hannover, Germany; 6 Institute of Virology, University Medical Center, Faculty of Medicine, University of Freiburg, Freiburg, Germany; 7 CAPNETZ Stiftung, Hannover, Germany; 8 Clinic of Respiratory Medicine, Medical Center, Faculty of Medicine, University of Freiburg, Freiburg, Germany

**Keywords:** *Streptococcus pneumoniae*, Serotypes, Serotype-specific urinary antigen detection, ssUAD, Community-acquired pneumonia, Switzerland

## Abstract

**Background:**

*Streptococcus pneumoniae* remains the leading bacterial cause of community-acquired pneumonia (CAP) and a major source of morbidity and mortality in adults. Pneumococcal conjugate vaccines (PCVs) have substantially reduced invasive pneumococcal disease, but serotype replacement has led to shifts in pneumococcal CAP epidemiology. In addition, the identification of *S. pneumoniae* remains challenging. This study investigated pneumococcal serotype distribution, vaccine coverage, and the diagnostic yield of a serotype-specific urinary antigen detection assay (ssUAD) among adults with CAP in Switzerland between 2016 and 2021.

**Methods:**

Adult patients enrolled in the Swiss CAPNETZ cohort with available urine samples were analyzed using the Pfizer 24-serotype ssUAD assay. Results were compared with conventional diagnostic methods, including the pneumococcal urinary antigen test (pUAT) and cultures. Serotype distribution, vaccine coverage (PCV13, PCV15, PCV20 and PCV21), and temporal trends were assessed.

**Results:**

Among 234 CAP patients, *S. pneumoniae* was identified by conventional diagnostics (pUAT and cultures combined) in 37 (15.8%). The ssUAD was positive in 35 patients (15.0%). Among patients with available results from both conventional diagnostics and ssUAD (*n* = 161), the addition of ssUAD increased pneumococcal CAP detection from 18.6% to 29.2% (*p* = 0.036). Among ssUAD-positive patients, the most frequent serotypes detected by ssUAD were 3 (*n* = 12), 8 (*n* = 5), and 11A (*n* = 4). Based on ssUAD-derived serotype distribution, vaccine coverage was 54.3% for PCV13, 60.0% for PCV15, 91.4% for PCV20, and 85.7% for PCV21. During 2020–2021, the proportion of serotypes not covered by PCV13 increased from 10/28 (35.7%) to 6/7 (85.7%) (*p* = 0.018).

**Conclusions:**

The addition of ssUAD improved *S. pneumoniae* detection beyond standard methods. Although based on a small number of cases, the shift toward non-PCV13 serotypes underscores the potential benefit of higher-valency vaccines such as PCV20 and PCV21 for adult pneumococcal disease prevention in Switzerland.

**Supplementary Information:**

The online version contains supplementary material available at https://doi.org/10.1186/s12879-026-14493-y.

## Introduction

Community-acquired pneumonia (CAP) is a common and lethal infection, significantly impacting healthcare systems on a global scale, causing approximately 3 million deaths each year [1]. *Streptococcus pneumoniae* continues to be the predominant bacterial pathogen identified in CAP worldwide [2], accounting for 10–15% of pneumonias [3]. Pneumococcal pneumonia, particularly prevalent among the elderly, exhibits high incidence and mortality rates [4]. In Switzerland, the annual incidence of CAP requiring hospitalization was reported to be 536 cases per 100,000 inhabitants between 2011 and 2015, with approximately 5% attributed to pneumococcal CAP [5]. In addition to non-invasive pneumococcal pneumonia, *S. pneumoniae* can cause invasive pneumococcal disease (IPD), defined by isolation of the pathogen from normally sterile body sites. In Switzerland, approximately 550–950 cases of IPD are diagnosed annually, of which around three quarters are due to pneumonia. Approximately 100 individuals die each year from IPD, with around 80% of deaths occurring among individuals older than 65 years [6].

Pneumococcal conjugate vaccines (PCVs), initially developed as pediatric vaccines, have significantly reduced IPD across all age groups globally through herd protection effects [7, 8] In Switzerland, the heptavalent pneumococcal conjugate vaccine (PCV7, Prevenar^®^), which covers serotypes 4, 6B, 9V, 14, 18C, 19F, and 23F, was first recommended in 2001 for children under 5 years old with an elevated risk for IPD [9]. In 2005, PCV7 was additionally recommended as an optional vaccination for healthy children under 2 years of age. The thirteen-valent pneumococcal conjugate vaccine (PCV13, Prevenar13^®^), which includes six additional serotypes (1, 3, 5, 6A, 7F, and 19A), replaced PCV7 in 2010. By 2014, PCV13 became the sole recommended pneumococcal vaccine, replacing the 23-valent pneumococcal polysaccharide vaccine (PPV23, Pneumovax23^®^). Following the implementation of PCVs, the pneumococcal serotype distribution has evolved towards replacement serotypes that are not covered by current vaccines and thus the serotype coverage of PCVs has decreased over time [9–11].

Since January 2024, higher-valency PCVs have been recommended for adults 65 years or older or with certain comorbidities. Since June 2024, a 15-valent PCV (PCV15, Vaxneuvance^®^) including also serotypes 22F and 33F, and a 20-valent PCV (PCV20, Prevenar20^®^) including also serotypes 8, 10A, 11A, 12F and 15B are licensed and available in Switzerland. Most recently, from 2026, PCV21 (Capvaxive^®^), a new higher-valency conjugate vaccine designed to provide broader serotype coverage, has been available and recommended for adults aged ≥ 65 years and those with certain comorbidities.

Respiratory cultures are underutilized, whereas blood cultures have limited diagnostic yield in pneumococcal CAP because they detect only cases with bloodstream invasion and are therefore frequently negative in non-invasive pneumococcal pneumonia, which constitutes the majority of pneumococcal disease. Furthermore, initial antibiotic treatment in outpatients may result in false-negative cultures. Consequently, most cases are detected using the pneumococcal urinary antigen test (pUAT; BinaxNOW *S. pneumoniae*) [12–14]. However, since the commercially available pUAT does not enable serotype discrimination, data on serotype distribution in adult non-bacteraemic pneumococcal CAP patients are limited [15].

For this study, we used a non-commercial serotype-specific urine antigen detection (ssUAD) assay on patients included in the Swiss CAPNETZ cohort study, a prospective observational multicenter cohort study of CAP patients. We analyzed the serotype distribution, temporal trends, outcomes, and the proportion of pneumonia cases caused by the serotypes included in PCV13, PCV15, PCV20, and PCV21 among adult patients with CAP in Switzerland between 2016 and 2021. We hypothesize that the ssUAD demonstrates high diagnostic performance for the identification of *S. pneumoniae* in adult patients CAP, showing good sensitivity and specificity compared to conventional microbiological methods. In addition, we aimed to verify whether among non-bacteraemic pneumococcal CAP cases identified by ssUAD, the proportion caused by PCV13 serotypes decreased from 2016 to 2021.

## Methods

All patients enrolled in the CAPNETZ study in Switzerland between 2016 and 2021 with an available urine sample were included in the analysis. The Swiss CAPNETZ cohort was initiated as a prospective, observational sub-study to collect data on CAP patients via the platform and eCRF of the German Competence network study of CAP (CAPNETZ, http://www.capnetz.de) with 39 sites in Europe, thereof 33 centers in Germany. Recruitment of the Swiss patients took place at two large tertiary care academic hospitals with diverse catchment areas in Switzerland between 2010 and 2022 (i.e. University Hospital Basel and Cantonal Hospital St. Gallen). CAPNETZ inclusion criteria were, as previously reported [16], age ≥ 18 years, radiologically confirmed pulmonary infiltrate, and at least one of the following clinical findings: cough, purulent sputum, fever or focal chest signs on auscultation. Up to 31 December 2019, exclusion criteria were hospitalization during the 28 days preceding the study, immunosuppression, and active tuberculosis. Since 2020 immunosuppression (ongoing pharmacological immunosuppression including chronic systemic steroid therapy, following transplantation, or due to rheumatological or other underlying diseases, chemotherapy, congenital or acquired immunodeficiency syndromes, active hematological neoplasia, or neutropenia) and SARS-CoV-2 infection were not further considered as exclusion criteria. The study was conducted in accordance with the Declaration of Helsinki. All patients provided written informed consent prior to enrolment for the study, and the study was approved by the local ethical committees of each participating center (Ethikkommission Nordwest- und Zentralschweiz EKNZ and Ethikkommission Ostschweiz EKOS, BASEC ID 2009 − 296).

Conventional diagnostic testing, including the pneumococcal urinary antigen test (pUAT) and microbiological cultures (blood, sputum, and bronchoalveolar lavage [BAL]), was performed at the discretion of the treating physicians (Supplemental Table 1).

A direct comparison of diagnostic yield between ssUAD and conventional methods was performed in a predefined subset of patients who underwent both ssUAD and full conventional diagnostic testing, defined as pUAT and at least one culture from blood, sputum, or BAL.

### ssUAD

Urine samples of enrolled patients were prospectively collected. Specimens were processed at the local laboratory and shipped to Pfizer’s Vaccines Research and Development Laboratory (Pearl River, NY, USA) where the UAD assays were performed in batch. The Pfizer serotype-specific urinary antigen detection assay is a limit assay that uses Luminex technology to detect multiple *S. pneumoniae* serotypes in a single human urine sample [17, 18]. The UAD-1 assay detects the serotypes contained in PCV13, while the UAD-2 assay detects 11 additional serotypes (the seven additional included in PCV20, i.e. 8, 10A, 11A, 12F, 15B, 22F and 33F, and the four included in PPV23, i.e. 2, 9N, 17F and 20). For every patient, the CAPNETZ investigators assessed available culture and pUAT results to determine pneumonia etiology. ssUAD results were not available at the time of this assessment.

No serotyping of culture-positive *S. pneumoniae* isolates was performed in this study; serotype determination relied exclusively on ssUAD. In Switzerland, routine serotyping is mandatory only for invasive pneumococcal isolates and is conducted by the Swiss National Reference Center for Invasive Pneumococci; these data were not available for the present study.

### Statistical analysis

Continuous variables are reported as medians with corresponding interquartile ranges (IQR), while categorical variables are expressed as frequencies and percentages within each specified group. Group comparisons were conducted using the Chi-square test for categorical variables, the t-test for comparing means, and the Mann-Whitney U test for comparing medians. Agreement between ssUAD and conventional microbiological testing (as defined above) was assessed using Cohen’s kappa coefficient; differences in paired positivity rates were evaluated using McNemar’s test. Statistical significance was determined by a two-sided p-value of less than 0.05. Statistical analyses were performed using SPSS Statistics, version 28, software (IBM, Armonk, NY, USA).

## Results

A total of 234 patients were included in the study, of whom 84 (35.9%) were female. The mean age was 62.8 years, and 97.9% were hospitalized (Table 1). A total of 186 patients (79.5%) were enrolled after the 2020 study amendment.

### Pneumococcal CAP vs. non-pneumococcal CAP according to standard of care testing

**Fig. 1:**
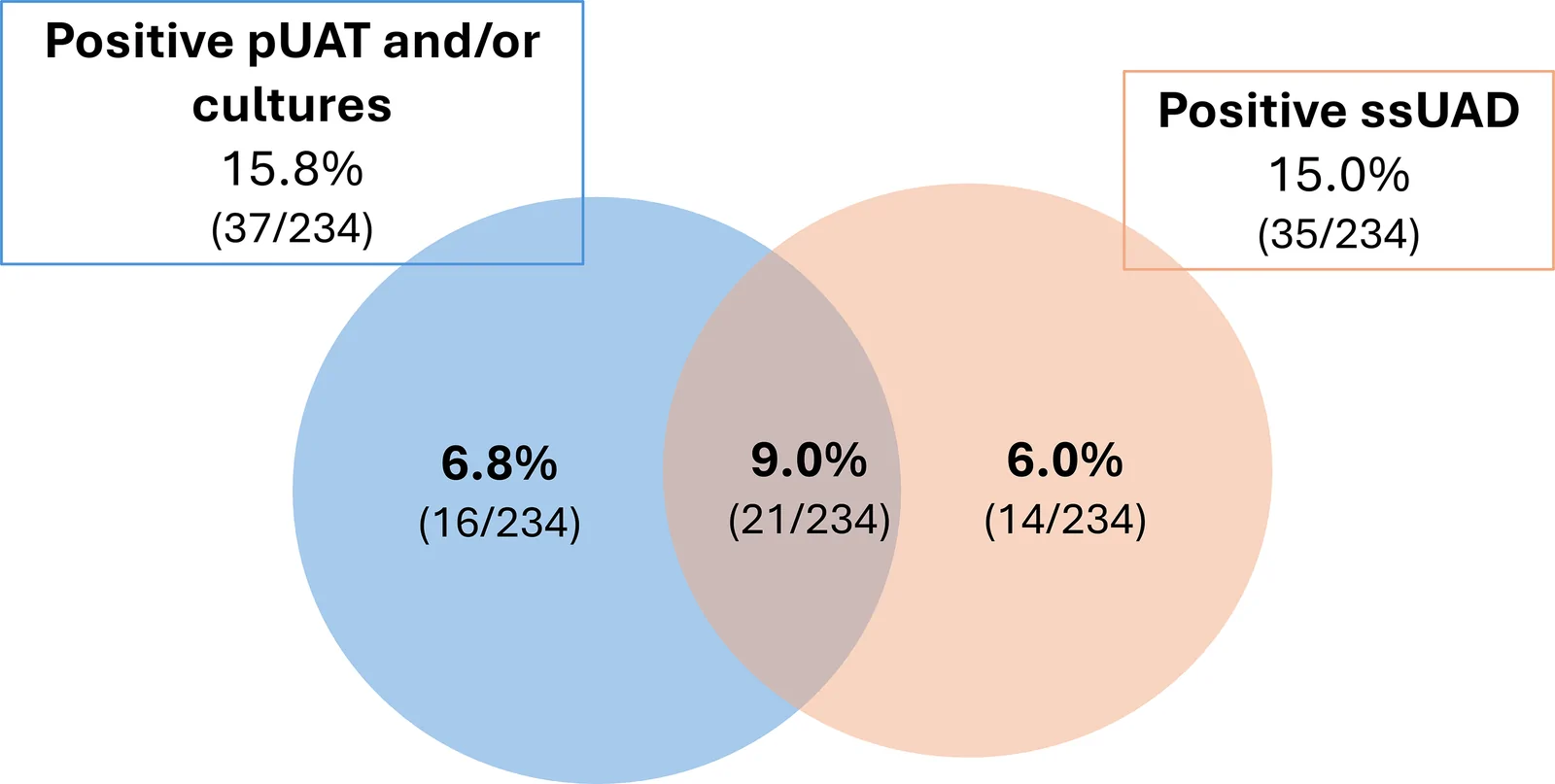
Comparison of pneumococcal CAP cases identified by conventional methods and ssUAD in the entire study cohort. Conventional methods included pUAT and/or ≥ 1 positive culture (blood, sputum, or BAL) for *Streptococcus pneumoniae*. All patients (*n* = 234) underwent ssUAD testing and at least one conventional test, but not all received both pUAT and cultures

*S. pneumoniae* was identified in 37/234 patients (15.8%) based on conventional diagnostic tests (pUAT, and/or blood, sputum and BAL cultures). Among these 37 patients, 21 (56.8%) also had a positive ssUAD result, while 16 (43.2%) were ssUAD-negative (Fig. 1).

### Pneumococcal CAP according to ssUAD

Overall, 35/234 patients (15.0%) had a positive ssUAD result. Of these, 21/35 (60.0%) were also identified by conventional diagnostics, whereas 14/35 (40.0%) were not detected by standard of care testing.

There was no significant difference in age between patients with positive and negative ssUAD test results (Table 1). The most frequently reported comorbidities were malignant disease (59/186 patients, 31.7%) and chronic lung diseases (74/234 patients, 31.6%), of which COPD was the most frequent disease (42/234 patients, 17.9%). Notably, none of the ssUAD-positive patients had received pneumococcal vaccination, compared to 7 of 199 patients (3.5%, *p* = 0.260) in the ssUAD-negative group. The median hospital stay was 7 days in both groups (IQR 4–10, *p* = 0.392). The ICU admission rate was higher in the ssUAD-positive group than in the ssUAD-negative group (8.6% vs. 4.5%), though this difference did not reach statistical significance (*p* = 0.317). There was no death at 180 days in the ssUAD-positive group; mortality rates in the ssUAD-negative group were 4.0% at 28 days and 6.5% at 180 days (*p* = 0.227 and *p* = 0.120).

### PCV13 CAP vs. NON-PCV13 CAP

There was no significant difference in age between patients with positive ssUAD results for *S. pneumoniae* serotypes covered by the PCV13 vaccine and those with non-PCV13 serotypes, although patients with PCV13 serotypes tended to be older (mean age: 67.7 years vs. 59.4 years, *p* = 0.096; Table 1). Furthermore, patients with PCV13 serotypes had a similar median hospital stay (8 days [IQR 5–13] vs. 7 days [IQR 4–8], *p* = 0.203) and a similar ICU admission rate (10.5% vs. 6.3%, *p* = 0.653).

**Table 1:** Baseline characteristics and outcomes of enrolled patients. Group comparisons were conducted using the Chi-square test for categorical variables, the t-test for comparing means, and the Mann-Whitney U test for comparing medians

	All CAP (*n* = 234)	ssUAD status			Serotype among ssUAD-positive patients		
		Negative (*n* = 199)	Positive (*n* = 35)	*p*-value	PCV13 (*n* = 19)	NON-PCV13 (*n* = 16)	*p*-value
Female – n (%)	84 (35.9)	74 (37.2)	10 (28.6)	*0.327*	5 (26.3)	5 (31.3)	*0.748*
Mean age - years	62.8	62.6	63.9	*0.657*	67.7	59.4	*0.096*
Median age - years (IQR)	66	66 (52–74)	68 (59–73)	*0.291*	69 (62–73)	63 (50–75)	*0.229*
Patients in 18–65 years age group – n (%)	112 (47.9)	99 (49.7)	13 (37.1)	*0.169*	6 (31.6)	7 (43.8)	*0.458*
Patients in ≥ 66 years age group – n (%)	122 (52.1)	100 (50.3)	22 (62.9)	*0.169*	13 (68.4)	9 (56.3)	*0.458*
Chronic lung disease – n (%)	74 (31.6)	59 (29.6)	15 (44.1)	*0.121*	9 (47.3)	6 (37.5)	*0.557*
*COPD* – n (%)	42 (17.9)	32 (16.1)	10 (28.6)	*0.076*	6 (31.6)	4 (25.0)	*0.668*
*Asthma* – n (%)	19 (8.1)	14 (7.0)	5 (14.3)	*0.148*	3 (15.8)	2 (12.5)	*0.782*
*Bronchiectasis* – n (%)	10 (4.3)	10 (5.0)	0 (0)	*0.175*	0 (0)	0 (0)	*-*
*Pulmonary fibrosis* – n (%)	1 (0.4)	1 (0.5)	0 (0)	*0.674*	0 (0)	0 (0)	*-*
*Ventilation* – n (%)	2 (0.9)	2 (1.0)	0 (0)	*0.551*	0 (0)	0 (0)	*-*
Congestive heart failure – n (%)	34 (15.6)	30 (15.1)	4 (11.4)	*0.572*	2 (10.5)	2 (12.5)	*0.855*
Diabetes – n (%)	40 (17.1)	35 (17.6)	5 (14.3)	*0.632*	2 (10.5)	3 (18.8)	*0.489*
Severe immunosuppression – n/total n (%)*	22/186 (11.8)	20/160 (12.5)	2/26 (7.7)	*0.481*	1 (5.3)	1 (6.3)	*0.9*
Malignant disease – n/total n (%)*	59/186 (31.7)	54/160 (33.75)	5/26 (19.2)	*0.140*	3 (15.8)	2 (12.5)	*0.782*
Chronic kidney disease – n (%)	50 (21.4)	41 (20.6)	9 (25.7)	*0.496*	6 (31.6)	3 (18.8)	*0.387*
Chronic liver disease – n (%)	5 (2.1)	3 (1.5)	2 (5.7)	*0.112*	1 (5.3)	1 (6.3)	*0.9*
Patients with positive pneumococcal vaccination status – n (%)	7 (3.0)	7 (3.5)	0 (0)	*0.260*	0 (0)	0 (0)	*-*
Inpatients – n (%)	229 (97.9)	196 (98.5)	33 (94.3)	*0.112*	17 (89.5)	16 (100)	*0.339*
Inpatients’ median length of stay – days (IQR)	7 (4–10)	7 (4–10)	7 (4–10)	*0.392*	8 (5–13)	7 (4–8)	*0.203*
Patients admitted to ICU – n (%)	12 (5.1)	9 (4.5)	3 (8.6)	*0.317*	2 (10.5)	1 (6.3)	*0.653*
Patients deceased at 28 days - n (%)	8 (3.4)	8 (4.0)	0 (0)	*0.227*	0 (0)	0 (0)	*-*
Patients deceased at 180 days – n (%)	13 (5.6)	13 (6.5)	0 (0)	*0.120*	0 (0)	0 (0)	*-*

Legend: *: information available only in patients included after the 2020 study amendment

### Comparison of ssUAD to other microbiological tests

pUAT was performed in 172/234 patients (73.5%) and was positive in 22 (12.8%). Sputum, BAL, and blood cultures were performed in 44 (18.8%), 92 (39.3%), and 200 (85.5%) patients, respectively. *S. pneumoniae* growth was detected in 6/44 sputum cultures (13.6%), 2/92 BAL fluid cultures (2.2%), and 7/200 blood cultures (3.5%). Although some patients underwent culture testing of more than one specimen type, *S. pneumoniae* was detected in only one specimen type per patient. Accordingly, the overall detection rate of *S. pneumoniae* by any culture method was 15/201 patients (7.5%). Among ssUAD-positive patients who also underwent additional conventional testing, concordance with conventional methods was limited. A positive pUAT result was observed in 13/29 ss-UAD-positive patients (44.8%). *S. pneumoniae* was identified in 7/33 (21.2%) by blood culture and in 2/7 (28.6%) by sputum culture, while none of the 8 patients tested by BAL (0%) had a positive result (Supplement Table 2).

**Fig. 2:**
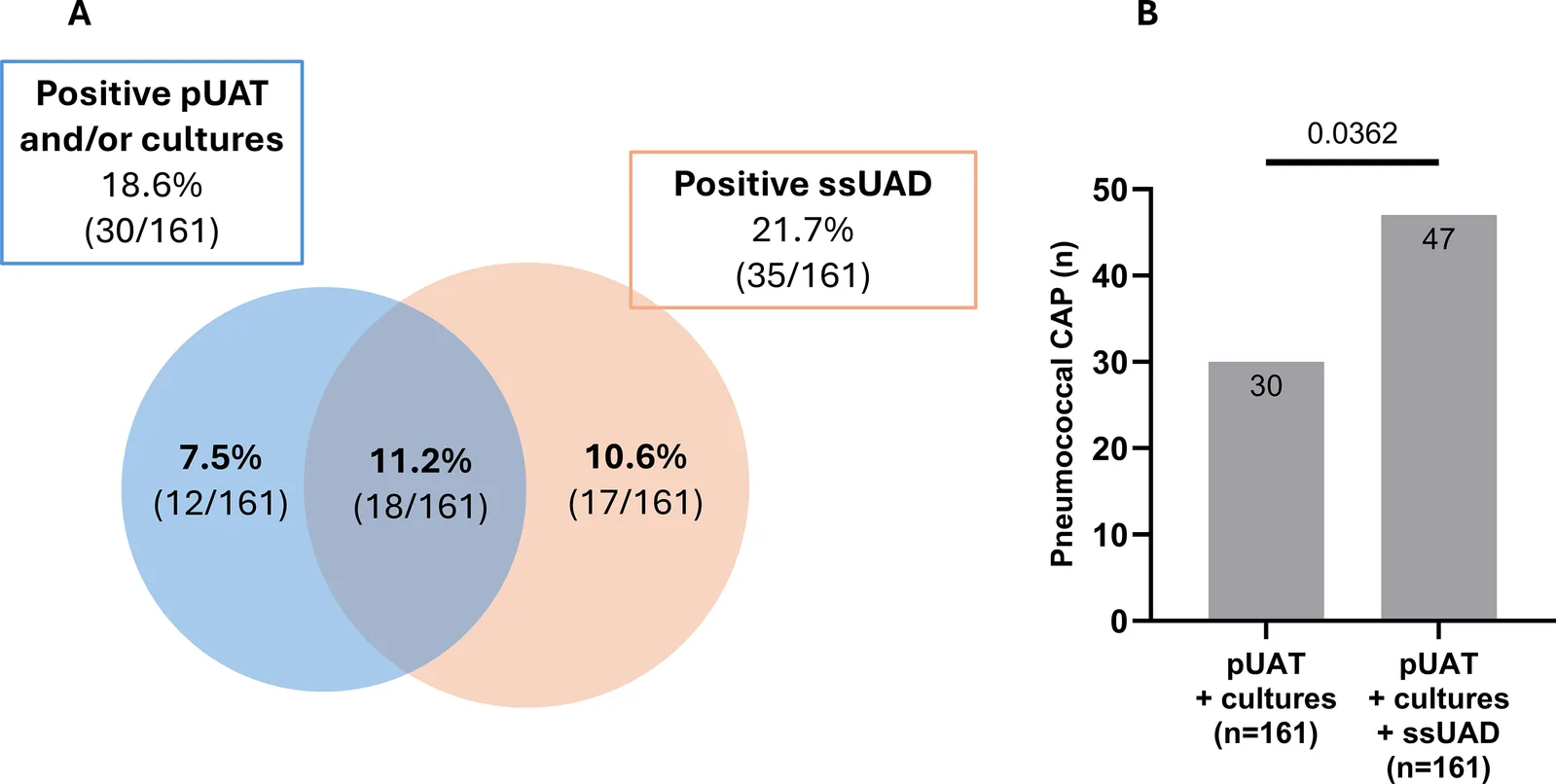
Direct comparison of the diagnostic yield of ssUAD and conventional methods. Conventional methods included the pneumococcal urinary antigen test (pUAT) and ≥ 1 culture from blood, sputum, or bronchoalveolar lavage (BAL). The analysis was restricted to patients who underwent ssUAD and full conventional testing (pUAT and ≥ 1 culture; *n* = 161). (**A**) Venn diagram showing the percentage and number or patients with positive ssUAD, positive conventional methods and their overlap. (**B**) Comparison of the number of pneumococcal CAP cases identified in this subset by pUAT and/or culture only compared with the combined use of pUAT, culture, and ssUAD

In the subset of 161 patients who underwent ssUAD in combination with full conventional diagnostic testing (pUAT and ≥ 1 culture), 30 were diagnosed with pneumococcal CAP using conventional methods alone (Fig. 2A). The addition of ssUAD increased the number of identified cases to 47 (Fig. 2B). Agreement between ssUAD and conventional microbiological testing (pUAT plus culture-based methods) was moderate (Cohen’s κ = 0.442, 95% CI 0.255–0.608). McNemar’s test showed no significant difference in paired positivity rates between the two diagnostic approaches (*p* = 0.458).

### Serotype distribution and vaccine coverage

**Fig. 3:**
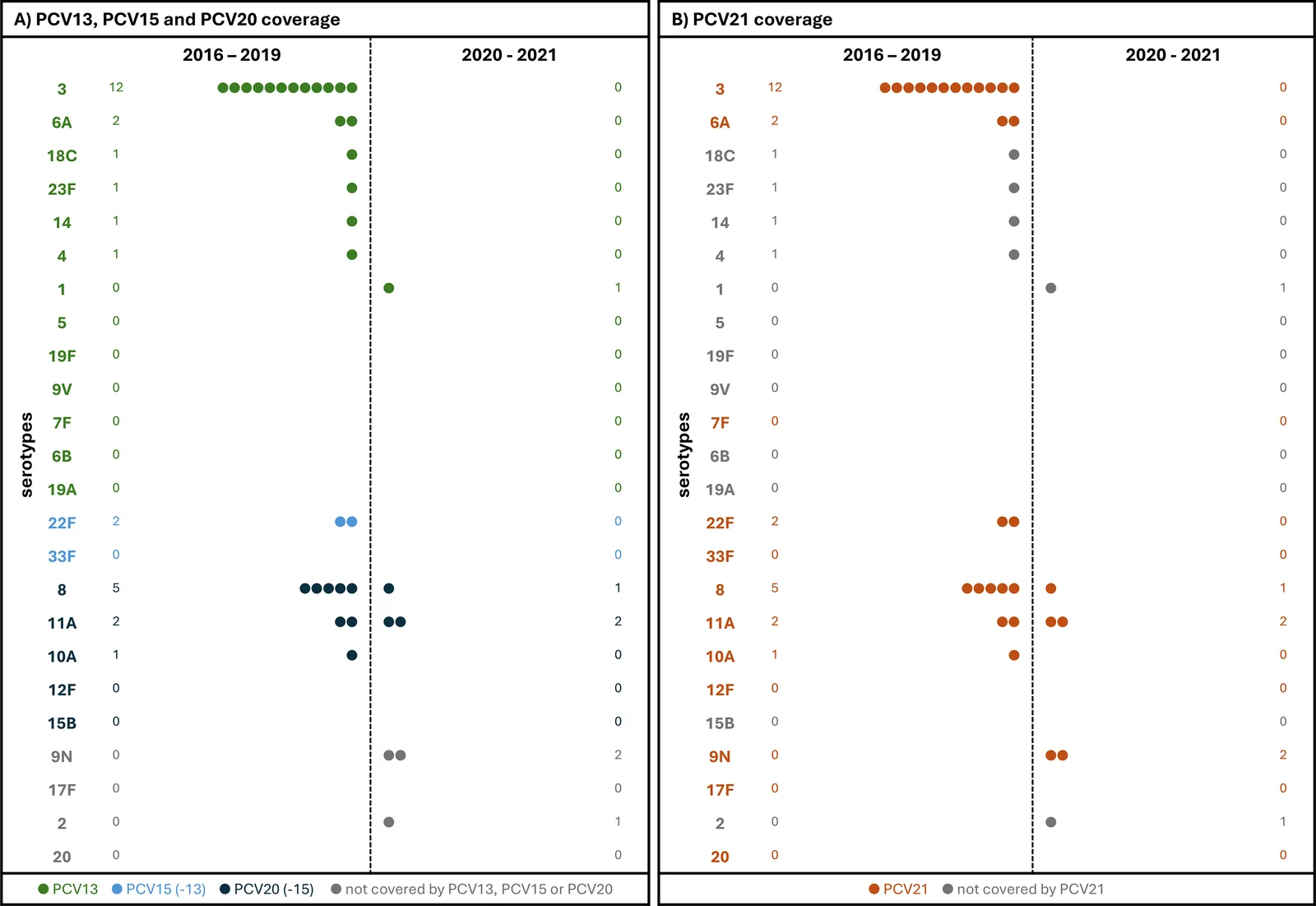
Serotype distribution and vaccine coverage for 2016–2019 and 2020–2021. Each dot represents one patient. (**A**) Green indicates PCV13 serotypes; light blue, additional PCV15 serotypes; dark teal, additional PCV20 serotypes. Grey indicates serotypes not covered by PCV13, PCV15, or PCV20. (**B**) Orange indicates serotypes covered by PCV21; grey indicates non-covered serotypes. Note: Some PCV21 serotypes were not assessed by the Pfizer ssUAD assay

The most frequently identified serotypes were serotype 3 (12/35, 34.3%), serotype 8 (6/35, 17.1%), and serotype 11A (4/35, 11.4%). Serotypes covered by PCV13 were identified in 19 patients (54.3%). An additional 2 patients (5.7%) had serotypes exclusively covered by PCV15, and 11 patients (31.4%) had serotypes included only in PCV20. In total, 21/35 patients (60.0%) had serotypes covered by PCV15, and 32/35 (91.4%) by PCV20 (Fig. 3A). PCV21 would have covered 30/35 patients (85.7%), including 3 patients (8.6%) with serotypes not included in PCV20 (Fig. 3B).

### Evolution during the COVID-19 pandemic

Between 2016 and 2019, *S. pneumoniae* was identified in 28/157 patients (17.8%). During 2020–2021, it was detected in 12/77 patients (15.6%). The proportion of positive ssUAD results showed a non-significant decrease from 28/157 (17.8%) in 2016–2019 to 7/77 (9.1%) in 2020–2021 (*p* = 0.078). The proportion of serotypes not covered by PCV13 increased from 10/28 (35.7%) to 6/7 (85.7%) (*p* = 0.018). In 2016–2019, serotypes 3 and 8 were most frequently identified. In 2020–2021, serotypes 11A and 9N were the most frequently detected, each being identified in 2/7 patients (Fig. 3).

## Discussion

This study investigated the distribution of *S. pneumoniae* serotypes among adults with CAP in Switzerland using the Pfizer ssUAD. *S. pneumoniae* was the most frequently identified pathogen using conventional diagnostic methods (pUAT and cultures), accounting for 15.8% of CAP cases, consistent with previous reports [19]. The addition of ssUAD to conventional diagnostic methods increased the detection of pneumococcal CAP. A shift towards non-PCV13 serotypes was observed during the 2020–2021 period compared with 2016–2019.

The ssUAD demonstrated a positivity rate of 15.0%, closely matching the 15.8% detection rate achieved with combined conventional microbiological testing. Although limited to 24 pneumococcal serotypes, ssUAD showed a 17% higher diagnostic yield than pUAT alone and increased the detection of *S. pneumoniae* by 10.6% (56.7% relative increase) when added to conventional methods. Agreement between ssUAD and conventional testing was moderate, with fewer than half of ssUAD-positive patients also testing positive by pUAT. Using the composite reference of pUAT and culture-based methods, ssUAD identified 60.0% of conventionally detected pneumococcal cases, indicating that neither approach alone captures all cases. This incomplete concordance is expected because the methods target different aspects of pneumococcal infection. Blood cultures are only positive in bacteraemic disease, respiratory cultures are limited by prior antibiotic exposure and specimen quality, and pUAT lacks serotype information. In contrast, ssUAD detects pneumococcal serotypes directly from urine, independent of bacterial viability or bloodstream invasion, but only for a limited number of serotypes. Clinical validation studies have demonstrated excellent diagnostic performance of the assay, with sensitivities of 97% for UAD1 and 92.2% for UAD2 and specificities of 100% and 95.9%, respectively [17, 18]. Consistent with our findings, previous studies have shown that ssUAD identifies substantially more cases of pneumococcal CAP than blood cultures and more than pUAT alone, while providing unique diagnoses not detected by conventional methods [20–24]. Although primarily developed as a research tool, these findings support the value of ssUAD as a complementary diagnostic assay for improving pneumococcal detection and enabling serotype-specific surveillance.

Several studies have reported a decline in invasive pneumococcal disease (IPD) during the COVID-19 pandemic, likely associated with public health measures such as social distancing, mask use, hand hygiene, travel restrictions, quarantine, isolation, and limiting large gatherings [25–28], and it is postulated that the control of respiratory viruses by these measures may have contributed to the decrease in IPD cases [26, 27]. In the present cohort, the proportion of pneumococcal CAP identified by cultures or pUAT remained stable during the COVID-19 pandemic compared to the 2016–2019 period, even if *S. pneumoniae* was surpassed by SARS-CoV-2 as the predominant pathogen during the pandemic. However, it must be stressed that this study was based on a convenience sample and does not allow conclusions regarding incidence trends. The proportion of patients with a positive ssUAD result decreased during the pandemic (9.1% vs. 17.8%), which may reflect changes in circulating serotypes, although the sample size was limited and serotype data were not available for ssUAD-negative but culture- or pUAT-positive cases.

A shift towards non-PCV13 serotypes was observed during the 2020–2021 period. This finding should be interpreted with caution, as it is based on only seven ssUAD-positive patients. Notably, neither serotype 3 nor serotype 8 were detected during this period, although both were among the predominant serotypes before the pandemic and caused high proportions of IPD in Switzerland during 2020 and 2021 [29]. Their apparent disappearance is most likely attributable to the limited sample size rather than a true epidemiological change. In Switzerland, IPD incidence declined markedly during 2020 before increasing again in early 2021, while serotype replacement continued with an increasing proportion of non-PCV13 serotypes [9, 29]. Similar patterns were reported across Europe, where overall IPD incidence decreased by approximately 60–80% during the COVID-19 pandemic, yet serotypes 3 and 8 consistently remained among the predominant causes of adult IPD [30–33]. In England, serotype 3 subsequently became the most prevalent IPD serotype following the relaxation of pandemic restrictions [34]. Genomic surveillance studies have suggested that the continued prominence of serotypes 3 and 8 may partly reflect the expansion of successful lineages with enhanced virulence and immune-evasion characteristics, including the CC180 lineage for serotype 3 and the CC53/GPSC3 lineage for serotype 8 [35–37]. Conversely, the increasing proportion of non-PCV13 serotypes observed in our cohort is consistent with long-term serotype replacement following PCV implementation and mirrors trends reported in Swiss surveillance, where the proportion of non-PCV13 serotypes increased from 59.1% in 2015 to 68.7% in 2023 [9, 29], as well as observations from Spain and Canada describing an increasing contribution of non-PCV13 serotypes to IPD during and after the COVID-19 pandemic [38, 39].

The field is largely moving towards serotyping of non-invasive pneumococcal pneumonia performed through ssUAD. Only few recent studies have used partial or complete data from serotyping of sputum isolates. A Japanese study used Quellung reaction from sputum but did not report ssUAD data and showed a predominance of serotypes 3, 35B, 15A, 11A and 23A, and PV20 serotype coverage of 44.1% [40]. A study of largely HIV-infected adults with pneumonia from South Africa demonstrated a concordance rate of 81% between a 13-serotype ssUAD and serotypes identified from respiratory specimens by Quellung and PCR [41]. Similarly, a US study reported that ssUAD identified all 14 PCV13 serotypes from culture specimens [15]. In contrast, a Canadian study confirmed a nearly equivalent serotype distribution from bacteremic and non-bacteremic pneumococcal pneumonia for some serotypes (e.g. serotypes 22F and 33F) but predominant identification through the ssUAD for other serotypes (e.g. 11A, 10A, 9N and 17F) [42]. These data support the value of ssUAD for serotype surveillance of non-invasive pneumococcal pneumonia.

This study has several limitations. The sample size was small and derived from 2 Swiss hospitals, limiting the assessment of serotype-specific trends and the generalizability of the findings. Rates of conventional diagnostic testing were not similar between the two participating centers, and differences in testing thresholds may have influenced the diagnostic yield of conventional methods. Selection bias may have occurred due to the requirement for informed consent, potentially underrepresenting critically ill patients. In addition, pneumococcal CAP may have been underestimated due to the limited serotype coverage of the ssUAD and the reduced sensitivity of both ssUAD and pUAT in non-bacteremic pneumonia [17].

In conclusion, the combined use of ssUAD, pUAT and cultures may improve the detection of *S. pneumoniae* in both clinical and research settings. The apparent increase in non-PCV13 serotypes during 2020–2021 should be interpreted cautiously due to the small number of ssUAD-positive cases. Most serotypes detected by the ssUAD were covered by PCV20, and these serotypes are also included among the serotypes targeted by PCV21.

## Supplementary Information

Below is the link to the electronic supplementary material.

Supplementary Material 1

Supplementary Material 2

## Data Availability

The data supporting the findings of this study are available from CAPNETZ Stiftung upon reasonable request and subject to approval.

## Abbreviations

CAP: Community-acquired pneumonia
PCV: pneumococcal conjugate vaccine
pUAT: Pneumococcal urinary antigen test
ssUAD: Serotype-specific urinary antigen detection
