# Supplementary material for: Pneumococcal serotype distribution and diagnostic yield of serotype-specific urinary antigen detection in adults with CAP in Switzerland, 2016–2021: a prospective cohort study
Source: BMC Infect Dis. 2026 Sep 21;26:1696. doi: 10.1186/s12879-026-14493-y (PMC13599082; doi:10.1186/s12879-026-14493-y)
Supplement: Supplementary file 1 [file 12879_2026_14493_MOESM1_ESM.docx]

| Test | University Hospital Basel (n=180) | Cantonal Hospital St. Gallen (n=54) |
| --- | --- | --- |
| Blood culture, n (%) | 150 (83.3) | 50 (92.6) |
| Sputum culture, n (%) | 31 (17.2) | 13 (24.1) |
| BAL culture, n (%) | 90 (50.0) | 2 (3.7) |
| pUAT, n (%) | 125 (69.4) | 47 (87.0) |

**Supplemental table 1:** Number of conventional microbiological tests performed by participating study center. Abbreviations: BAL, bronchoalveolar lavage; pUAT, pneumococcal urinary antigen test.
