# Supplementary material for: Pneumococcal serotype distribution and diagnostic yield of serotype-specific urinary antigen detection in adults with CAP in Switzerland, 2016–2021: a prospective cohort study
Source: BMC Infect Dis. 2026 Sep 21;26:1696. doi: 10.1186/s12879-026-14493-y (PMC13599082; doi:10.1186/s12879-026-14493-y)
Supplement: Supplementary file 2 [file 12879_2026_14493_MOESM2_ESM.docx]

**Supplemental table 2**: Results of other microbiological diagnostic tests in patients with positive ssUAD test result and in patients with a positive pUAT test result. Abbreviations: pUAT, pneumococcal urinary antigen test; serotype-specific urinary antigen detection assay; BAL, bronchoalveolar lavage.

|  | Positive pUAT (n=22) | Positive ssUAD  (n=35) |  |  |  |  |
| --- | --- | --- | --- | --- | --- | --- |
|  |  | Overall | *PCV13 serotypes*  *(n=19)* | *PCV 15 (-PCV13) serotypes (n=2)* | *PCV 20 (-PCV15) serotypes*  *(n=11)* | *NON-PCV 20 serotypes*  *(n=3)* |
| Patients with a positive pUAT – n/total n (%) | - | 13/29 (44.8) | *8/19 (42.1)* | *0/2 (0)* | *4/7 (57.1)* | *1/1 (100)* |
| Patients with a blood culture result positive for *S. pneumoniae* – n/total n (%) | 4/21 (19.0) | 7/33 (21.2) | *5/19 (26.3)* | *1/1 (100.0)* | *1/10 (10)* | *0/3 (0)* |
| Patients with a sputum culture result positive for *S. pneumoniae* – n/total n (%) | 3/6 (50.0) | 2/7 (28.6) | *0/2 (0)* | *0/0 (0)* | *2/4 (50.0)* | *0/1 (0)* |
| Patients with a BAL culture result positive for *S. pneumoniae* – n/total n (%) | 0/5 (0) | 0/8 (0) | *0/4 (0)* | *0/0 (0)* | *0/3 (0)* | *0/1 (0)* |
| Patients with *S. pneumoniae* evaluated to be responsible for CAP according to CAPNETZ experts– n (%) | 22 (100.0) | 21 (60.0) | *11 (57.9)* | *1 (50.0)* | *7 (63.6)* | *2 (66.6)* |
| Patients with any culture positive for *S. pneumoniae* and/or positive pUAT | - | 16/30 (53.3) | *-* | *-* | *-* | *-* |
